# Plasma Interleukin-10: A Likely Predictive Marker for Hepatitis B Virus-Related Acute-on-Chronic Liver Failure

**DOI:** 10.5812/hepatmon.19370

**Published:** 2014-07-14

**Authors:** Ke Wang, Zhe-bin Wu, Yi-nong Ye, Jing Liu, Geng-lin Zhang, Yu-jie Su, Hong-liang He, Yu-bao Zheng, Zhi-liang Gao

**Affiliations:** 1 Department of Infectious Diseases, Third Affiliated Hospital of Sun Yat-sen University, Guangzhou, China; 2 Department of Infectious Diseases, Foshan Hospital of Sun Yat-sen University, Foshan, China

**Keywords:** Chronic Hepatitis B, Severe Exacerbation, Chronic Liver Failure, Cytokines, Interleukin-10

## Abstract

**Background:**

The pathogenesis of HBV-related acute-on-chronic liver failure (HBV-ACLF) is mainly based on a heightened immune-inflammatory reaction; however, the intimate underlying mechanism remains unclear.

**Objectives:**

The aim of the study was to explore potential key immune molecular targets that could serve as early predictive markers for HBV-ACLF.

**Patients and Methods:**

Twenty-seven patients with acute exacerbation of chronic hepatitis B (CHB) (defined by: alanine transaminase ≥ 20 ULN, total bilirubin ≥ 5 ULN, 40% < prothrombin time activity ≤ 60%) and without cirrhosis were divided into 18 cases which did not progress to HBV-ACLF (defined by: prothrombin time activity < 40% and development within four weeks of hepatic encephalopathy and/or ascites) and nine cases that developed HBV-ACLF. Nine healthy people defined the normal control group (NC). Interleukin-1β (IL-1β), IL-2, IL-4, IL-6, IL-8, IL-10, IL-12p70, TNF-α and IFN-γ protein levels were assayed by Cytometric Bead Array (CBA) in blood plasma. The ELISA method was applied to confirm IL-10 detection using the CBA method.

**Results:**

IL-4, IL-12p70 and IFN-γ were undetectable; IL-1β, IL-6, IL-8, IL-10 and TNF-α levels were significantly higher than in NC. Moreover, cytokines reached the highest levels in acute exacerbation of CHB, with the exception of IL-2 and IL-8. When comparing the HBV-ACLF patients prior to and at the time of ACLF diagnosis, IL-10 was the only cytokine that exhibited a significant decrease (P = 0.008). IL-10 concentrations were positively correlated to ALT levels (r = 0.711, P < 0.001).

**Conclusions:**

The assessment of plasma IL-10 levels in chronic hepatitis B acute exacerbation may provide an early predictive marker for progression to HBV-ACLF.

## 1. Background

Chronic hepatitis B virus (HBV) infection is a major health problem, especially in Asia. The clinical outcomes of HBV infection are extremely variable and includes self-resolving acute hepatitis, chronic hepatitis B carriers, acute-on-chronic liver failure (ACLF) cirrhosis and hepatocellular carcinoma (1). In China, more than 80% of ACLF incidence is caused by HBV infection (2). HBV-related ACLF (HBV-ACLF) is an increasingly recognized syndrome corresponding to an acute and severe exacerbation of liver function in patients with chronic HBV infection. The pathogenic mechanism of HBV-ACLF is not well understood. Despite the identification of important predisposing factors and prognostic markers, HBV-ACLF remains a disease associated with high mortality (3-5). The majority of studies using nucleoside analog therapy, bioartificial liver support systems and stem cell transplantation have not shown a significant improvement in long-term survival; as a result, larger future studies are needed in this area of research (6-8). Liver transplantation is the best method for treating HBV-ACLF, but liver donation resources remain limited. Therefore, there exists a real challenge for finding key pathogenic targets in the development of HBV-ACLF for preventing the disease.

The development process of HBV-ACLF can be divided into prior to and at the time of diagnosing HBV-ACLF as it corresponds to the early stage, progression stage and remission stage. Immunological changes are relevant in the development process of HBV-ACLF. Our preliminary studies suggested that there were high immune activities in vivo at the early stage of HBV-ACLF (9). Fujiwara et al.’s reports also showed that the use of corticosteroid therapy in the early phase of HBV-related liver failure could partly improve liver regeneration and reduce mortality rate; however, its efficacy remains limited (10, 11). These studies also indicated the presence of a high immune state in the early phase of HBV-ACLF. It is generally agreed that rapid diagnosis and management are of primary importance for allowing survival; however, the indicators that can determine the disease's progression remain unclear.

## 2. Objectives

The aim of the study was to explore potential key immune molecular targets that could serve as early predictive markers for HBV-ACLF.

## 3. Patients and Methods

### 3.1. Patients

Patients who had been referred to the Department of Infectious Diseases at the third affiliated hospital of the Sun Yat-sen University (Guangzhou, China) between May 2009 and December 2012 were recruited for this study. The preliminary screening included 189 patients who were admitted for the treatment of severe chronic hepatitis B (CHB) or CHB-related liver failure. We removed 162 patients who do not meet the research standards in the following order: 60 cases with liver cirrhosis, 38 cases with incomplete hospitalization for economy, 54 cases with liver failure at admission and 10 cases using artificial liver support systems or immunomodulatory drugs therapy. Finally, 27 patients with acute exacerbation (_ae_CHB) were selected for participation. Eighteen of these 27 _ae_CHB patients did not progress to acute-on-chronic liver failure (HBV-ACLF) (as defined below), thereby corresponding to the “_ae_CHB without ACLF” patients; nine developed ACLF (corresponding to the “HBV-ACLF” patients). These 9 HBV-ACLF patients were studied both prior to and at the time of ACLF (corresponding to the “_before_HBV-ACLF” and “atHBV-ACLF” subgroups). Nine healthy people were recruited for the normal control group (NC). Plasma samples were collected in the time frame of the study concerning the pathogenesis of CHB-related liver failure, a process supported by the National Science and Technology Major Project (No. 2008ZX10002007, No. 2012ZX10002007). The study was conducted according to the guidelines of the Declaration of Helsinki and was approved by the Human Ethics Committee of the Third Affiliated; Sun Yat-sen University. Written informed consent was obtained from each subject.

### 3.2. The Diagnostic Criteria of CHB Acute Exacerbation and HBV-ACLF

The adopted criteria for the present study corresponded to those proposed by Fujiwara et al. and to the recommended guidelines of the 2009 Asian Pacific Association for the Study of the Liver (APASL), with minor modifications (11, 12). Briefly, the CHB acute exacerbation criteria were as follows: duration of plasma hepatitis B surface antigen (HBsAg) presence more than six months before entry, alanine transaminase (ALT) greater than 20 ULN (normal values range between 3 and 35 UI/L), total bilirubin (T-Bil) greater than 5 ULN (normal values range between 4 and 24 µmol/L), prothrombin time activity (PTA) less than 60% of normal controls and no cirrhosis according to imaging examination (ultrasound or computed tomography or magnetic resonance). Patients with PTA less than 40% and/or an international normalized ratio (INR) higher than 1.5, and who experience complications within four weeks brought on by hepatic encephalopathy and/or ascites were defined as having HBV-ACLF. Clinical assessment and blood plasma were performed approximately every week (5 ± 2 days) according to the degree of disease progression. Anti-viral therapies, including lamivudine or entecavir, were prescribed according to HBV replication levels, economical condition and willingness of the patient. No patients received corticosteroids, stem cell transfusions, bioartificial liver support or other immune related treatments prior to the diagnosis of HBV-ACLF.

The participants were free of autoimmune liver diseases, bacterial infection, fungal infection and viral infection, including human immunodeficiency (HIV), hepatitis C virus (HCV), cytomegalovirus (CMV) and Epstein-Barr virus (EBV). Patients with recent exposure to drugs and chemical agentsor with recent heavy alcohol intake were ruled out.

### 3.3. The Determination of Circulating Inflammatory Cytokines Using Flow Cytometry

Plasma cytokines were measured serially by cytometric bead array (CBA) using a FACSC alibur flow cytometer LSR II (Becton Dickinson, CA, USA). In brief, nine bead populations with distinct fluorescence intensities had capturing antibodies specific for different cytokines. These bead populations could be resolved in the fluorescence channels of the flow cytometer. After the beads had been incubated with 50 µL of plasma, different cytokines or chemokines in the sample were captured by their corresponding beads. The cytokine captured beads were then mixed with phycoerythrin-conjugated detection antibodies to form sandwich complexes. Following incubation, washing and acquisition of fluorescence data, the results were generated in graphical format using the Becton, Dickinson and Company (BD) CBA software. The concentrations of inflammatory cytokines Interleukin (IL)-1β, IL-2, IL-4, IL-6, IL-8, IL-10, IL-12 p70, tumor necrosis factor (TNF)-α and Interferon (IFN)-γ were measured using a CBA kit (BD Pharmingen, CA, USA). The assay sensitivities of these nine cytokines were 48.4 fg/mL, 88.9 fg/mL, 144.4fg/mL, 68.4 fg/mL, 69.9 fg/mL, 13.7 fg/mL, 12.6 fg/mL, 67.3 fg/mL and 66.7 fg/mL, respectively (data were supplied by the manufacturer’s kit). The inter- and intra-assay coefficient variations (CVs) were less than 10% for all cytokine assays.

### 3.4. The Detection of IL-10 Level Using ELISA Assay

To validate the CBA results, the plasma IL-10 levels were further determined using the enzyme linked immunosorbent assay (ELISA) method (R&D Systems, Inc., MN, USA) according to the manufacturer’s protocols. The detection sensitivity of each ELISA kit was 3.9 pg/mL for IL-10. The results showed that there was no significant difference concerning the CBA and ELISA assay detected data (P = 0.930).

### 3.5. Serum HBV DNA Assay and Laboratory Tests of Liver Function

The viral load of HBV DNA in the serum samples was quantified by a quantitative polymerase chain reaction assay (Roche Diagnostics, Basel, Switzerland). The detection sensitivity of the polymerase chain reaction (PCR) assay was 100 IU/mL. Laboratory tests of liver function, such as ALT, T-Bil and PTA were performed using standard methods in a clinical laboratory.

### 3.6. Statistical Analysis

The Mann-Whitney rank sum test was used for assessing the differences between groups, because the plasma cytokine concentrations were not in a Gaussian distribution. Separate repeated analyses of variances (ANOVAs) were performed for the prior to and at time of diagnosis of HBV-ACLF. A paired chi-square test was used to assess the agreement between CBA and ELISA methods. Data for the different groups with baseline characteristics were compared using a Pearson chi-square test and ANOVAs. Results were expressed as medians (interquartile range, or ± SEM). All analyses were performed using the Statistical Package for Social Sciences (SPSS) software for Windows, Version 13.0 (SPSS Inc., IL, USA). Values of P < 0.05 were considered statistically significant.

## 4. Results

### 4.1. Patient Characteristics

The clinical characteristics of these subjects are presented in Tables 1 and 2. Six patients did not receive anti-viral treatment, seven patients received lamivudine (LAM) and 14 patients received entecavir (ETV). The starting date of the antiviral treatment was the date of diagnosis during hospitalization for 15 patients. Six patients had received anti-viral therapy one year or more prior to the hospitalization. Among the nine patients who progressed to HBV-ACLF, five patients died and four recovered within three months.

**Table 1.** Baseline Characteristics of Patients and Normal Controls ^a^

Parameters	NC (n = 9)	_ae_CHB without ACLF (n = 18)	_before_HBV-ACLF (n = 9)	P Value
**Age ** ^**b**^ **, y**	38 (34-46)	41 (26-62)	50 (23-64)	0.201
**Gender, Male/Female**	8/1	17/1	8/1	0.834
**ALT ** ^**b**^ **, IU/L**	23.0 (15-35)	1085.5 (729-2698)	749.0 (705-1138)	0.000
**T-Bil ** ^**b**^ **, µmol/L**	11.60 (5-23)	178.95 (127-572)	320.90 (169-671)	0.000
**PTA ** ^**b**^ **, No. (%)**	96 (87-103)	56 (48-59)	52 (41-56)	0.000
**INR ** ^**b**^ **, No. (%)**	1.02 (0.98-1.11)	1.50 (1.41-1.78)	1.57 (1.48-1.93)	0.000
**HBeAg (+/-)**	0/9	9/9	2/7	0.166
**HBV-DNA ** ^**b**^ **, log_10_IU/mL**	ND ^c^	7.09 (0-8.65)	5.47 (2.87-7.99)	0.234^ d^
**Antiviral Drug, LAM/ETV**	0/0	4/9	3/5	0.751^ d^

^a^ NC, _ae_CHB without ACLF, _before_HBV-ACLF represent normal control, CHB acute exacerbation without ACLF group, before diagnosis of HBV-ACLF group, respectively.

^b^ For age, ALT, T-Bil, PTA, INR and HBV DNA, the median (range) for each group is shown.

^c^ Abbreviation: ND, not detected.

^d^ represents the comparison between aeCHB without ACLF group and _before_HBV-ACLF group.

**Table 2.** Changes in Clinical Characteristics Between “Prior to” and “at the Time of” Diagnoses of HBV-ACLF (Nine Cases)

Case	Age, y	Sex	NAs	HBeAg (+/-)	HBV DNA, IU/mL	Before diagnosis of HBV-ACLF					At the time of diagnosis of HBV-ACLF					Duration time ^a^, days	outcome
						ALT, IU/L	T-Bil, µmol/L	PTA, %	INR	Complication	ALT, IU/L	T-Bil, µmol/L	PTA, %	INR	Complication		
**1**	50	Male	ETV	-	2.93 × 10E5	714	169	54	1.57	-	239	373	54	1.56	Ascites	7	Recovery
**2**	53	Female	LAM	-	9.67 × 10E7	780	173	51	1.55	-	118	447	53	1.6	Ascites	7	Recovery
**3**	39	Male	ETV	-	1580	714	398	52	1.54	-	68	559	31	2.48	Encephalopathy /Ascites	6	Death
**4**	54	Male	LAM	+	16600	705	423	56	1.48	-	45	231	46	1.75	Ascites	7	Recovery
**5**	40	Male	NU ^b^	+	7.54 × 10E7	749	321	41	1.93	-	80	358	34	2.31	Ascites	5	Death
**6**	64	Male	ETV	-	6.88 × 10E6	1044	260	53	1.55	-	410	441	25	3.04	Ascites	6	Death
**7**	63	Male	ETV	-	5.35 × 10E6	1138	260	52	1.57	-	395	440	28	2.98	Ascites	6	Death
**8**	23	Male	LAM	-	10400	775	411	45	1.76	-	85	343	36	2.14	Ascites	7	Recovery
**9**	35	Male	ETV	-	744	705	671	54	1.58	-	110	607	37	2.13	Ascites	7	Death

^a^ Time interval between the two subsequent samples in the same patients.

^b^ NU: No anti-viral drugs.

### 4.2. Comparison of the Plasma Cytokine Levels Betweenthe Different Groups

**Figure 1.**
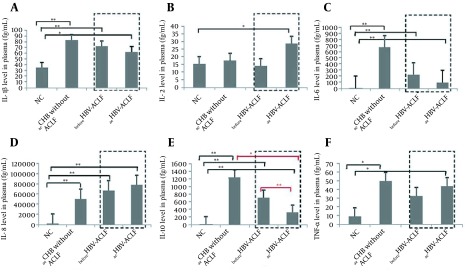
Comparison of IL-1β (A), IL-2 (B), IL-6 (C), IL-8 (D), IL-10 (E) and TNF-α (F) plasma levels in plasma between the four groups. Only IL-10 levels were significantly different between _ae_CHB without ACLF and the “before/at time of diagnosis” ACLF patients (P = 0.024). Dashed boxes display the plasma cytokine levels of _before_HBV-ACLF and atHBV-ACLF subgroups in the same patients. The data show that only IL-10 levels in atHBV-ACLF were significantly lower than _before_HBV-ACLF (P = 0.008). Data that are shown as mean ± SEM. P < 0.05 (*) and P < 0.01 (**) were considered to have significant differences between groups.

IL-4, IL-12p70 and IFN-γ levels were not detected (Figure 1). IL-1β, IL-6, IL-8, IL-10 and TNF-α levels were remarkably higher in all patient groups than in NC, except for TNF-α levels, which were not significantly different between ACLF patients prior to diagnosis and NC. When comparing the _ae_CHB without ACLF patients with HBV-ACLF patients (both before and at the time of ACLF diagnosis), IL-1β, IL-6, IL-10 and TNF-α levels were higher in the _ae_CHB without the ACLF group, but the only statistically significant difference concerned IL10 levels between the _ae_CHB without ACLF group and the *at time of* ACLF patients group (P = 0.024). When comparing the cytokine levels between the “before and at time of diagnosis” ACLF patients, the only significant difference was again related to the IL-10 levels, which were significantly lower at than before time of the diagnosis of ACLF (P = 0.008, Figure 1).

### 4.3. The Correlation of ALT Level and the Plasma Cytokine Level

**Figure 2.**
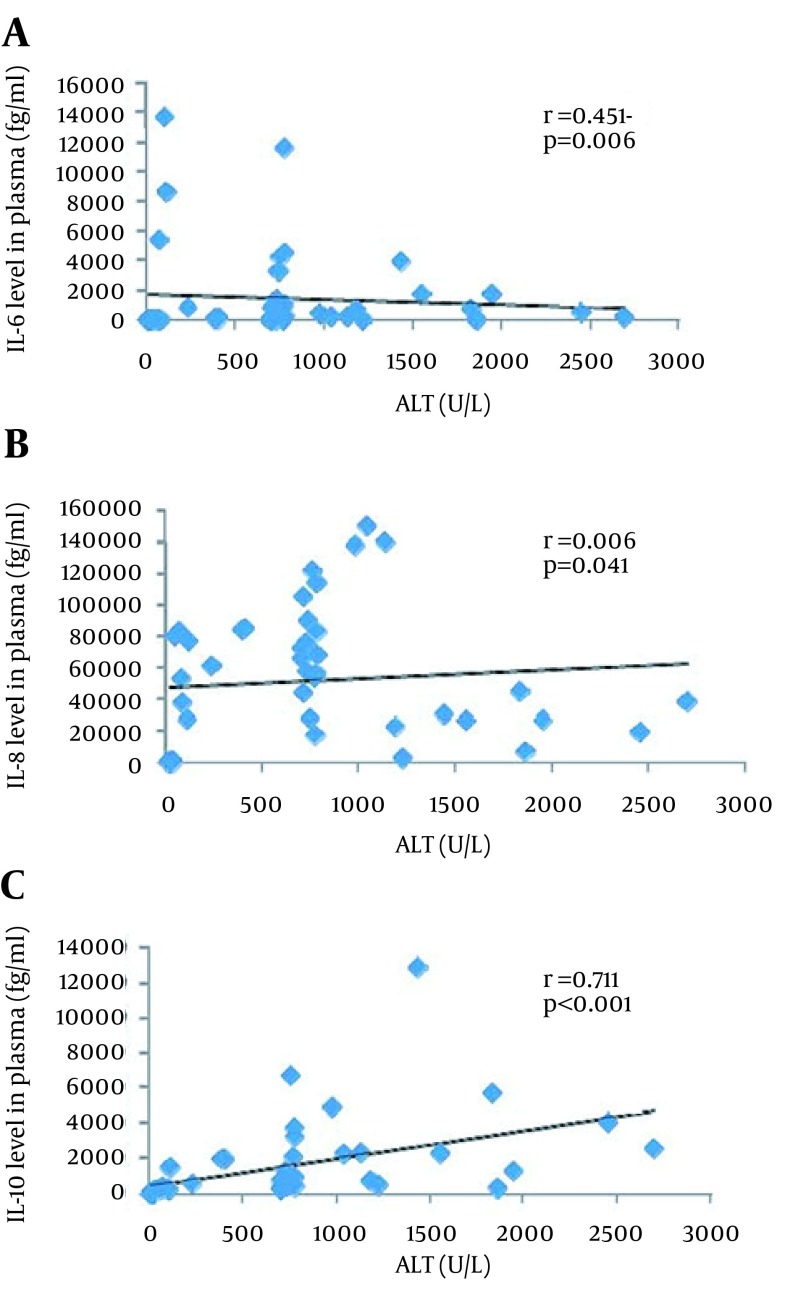
Correlations between the detected cytokines and ALT levels in the CHB patients and NC (n = 36) (IL-6 (A), IL-8 (B), and IL-10 (C)). The only convincing statistical correlation, when considering graphical representation, concerned IL-10.

To understand the possible relationship of ALT and immuno-inflammatory cytokines in the development of HBV-ACLF, we further analyzed the correlation between the ALT and the different cytokines levels. No significant correlations were observed forIL-1β, IL-2 and TNF-α. IL-6 6: r = 0.451, P = 0.006; for IL8: r = 0.342, P = 0.041), but the graphical representations did not confirm these trends. and IL-8 showed a theoretical statistical difference (for IL-In fact, the only significant correlation was for IL-10 with a high r value (0.711), a P value < 0.001 and graphical confirmation (Figure 2).

## 5. Discussion

It is has been well-established that acute exacerbation of CHB may lead to liver failure, with potential severe and even lethal consequences. An estimated 40-50% of hepatitis B e antigen-positive patients can undergo the immune clearance phase and partly develop CHB acute severe exacerbation (13). A three-month mortality in more than 50% of cases with HBV-ACLF without liver transplantation has been reported (7, 14). However, epidemiological data regarding the progression of _ae_CHB to HBV-ACLF are poorly documented. Our data showed that 33% of the patients progressed to HBV-ACLF and 56% of patients with HBV-ACLF died within three months. These results illustrate the unique severity of this disease, which should draw the attention of clinicians.

A number of studies have shown that the immune-inflammatory cytokines in the prophase or early phase of HBV-ACLF corresponds to a high-expression status, suggesting that cytokines play an important factor in the development of HBV-ACLF (9, 10, 15). In this context, we used the CBA method to detect the changes in the various cytokine levels in vivo prior to and at the time of the diagnosis of HBV-ACLF. Our preliminary results showed that IL-1β, IL-6, IL-10 and TNF-α levels in patients with _ae_CHB and without ACLF were higher than in the other groups and that IL-2 and IL-8 levels were higher at the time of HBV-ACLF. With the progression of the disease, IL-2 and IL-8, the two types of pro-inflammatory cytokines, increased continuously and may play an important role in promoting HBV-ACLF development. However, we found that the increase of IL-2 and IL-8 levels was not statistically different between the _ae_CHB without ACLF and the _before_HBV-ACLF groups. This may be related to the small number of samples and/or to the different efficacies of various anti-viral treatments, which may have interfered in the disease's progression; this isa point that requires further study. 

More specifically, we explored the dynamic changes of two cytokines, including IL-6 and IL-10 before ACLF and at time of ACLF diagnosis, with the hypothesis that the decline of their levels could be another key factor in promoting the progression of the disease. IL-10 level diminution, in contrast to IL-6 data, was significantly more pronounced at the time of HBV-ACLF than before HBV-ACLF. Several reports have shown that there was a differential expression of IL-10 levels in various disease states of chronic HBV infection. By studying the expression of intrahepatic cytokines through immunochemistry, Zou et al. observed similar anti-inflammatory IL-10 expression in ACLF and CHB (16). Hu et al. reported that IL-10 secretion by peripheral blood mononuclear cells stimulated by rhIL-21 was significantly increased in HBV-ACLF (17). Shen et al. reported that regulatory T cells were increased in HBV-ACLF and positively correlated with IL-10 levels (18). However, none of these studies were done in a longitudinal and dynamic manner, as was the case in the present study.

Considering that IL-10 is an important negative regulator of inflammation, this finding favors the close relationship between IL-10 levels and disease progression. Moreover, our results showed that IL-10 levels were significantly positively correlated to ALT levels (Figure 2). These data are consistent with that of Das et al. who reported that IL-10 levels were closely and positively related with HBV DNA load and ALT levels in the HBeAg-negative patients (19). In contrast, although IL-10 promoter polymorphisms and expression of IL-10 were closely related (20), Sofian et al. reported that IL-10 promoter polymorphisms were not correlated with HBV infection outcome in vivo (21). However, neither HBeAg- nor IL-10-level data were available in their study; therefore,these results remain difficult to explain. Interestingly, in two reported trials on hepatitis C, treatment with IL-10 has already been shown to result in normalization of aminotransferase levels, improved liver histology and reduced fibrosis (22, 23). Therefore, a new approach for preventing the progression of _ae_CHB disease to the HBV-ACLF could be to combine NAs antiviral drugs and IL-10 therapy. However, it should be noted that one of the two trials also reported that IL-10 therapy may increase viral replication.

Corticosteroids have been used in the treatment of chronic active hepatitis B since the 1980s (24). Currently, glucocorticoids are mainly used for acute exacerbation of CHB, but their specific duration and dose remain unclear (11, 15). Some experts have proposed that they may be applied to the early stage of HBV-ACLF (10); however, Karkhanis et al. reported that corticosteroids did not improve overall survival or spontaneous survival in drug-induced, indeterminate or autoimmune acute liver failure (25). Like corticosteroids, IL-10 is a negative regulator of inflammation and could therefore be considered as a possible therapeutic tool. In addition, our results suggest that negative regulatory treatment (corticosteroids or IL-10) should be started at the CHB acute exacerbation phase and not at the time of diagnosing liver failure. Regarding the correlation study between cytokines and ALT, our results support the fact that ALT levels could reflect, at least partly, the degree of inflammatory activity in vivo.

Studies larger in scope are warranted to confirm the present results, which indicate that a decrease inIL-10 levels may be one of the main factors associated with the pathogenesis of HBV-ACLF. As most cytokines (IL-1β, IL-6, IL-10, TNF-α) in this study reached the highest expression in the acute exacerbation group, immunosuppressive drugs or other negative regulators of immune status should preferably be introduced at this earlier stage. From a practical point of view, the assessment of plasma IL-10 levels in chronic hepatitis B acute exacerbation may provide an early predictive marker for progression to HBV-ACLF.

## References

[1] Liaw YF, Kao JH, Piratvisuth T, Chan HLY, Chien RN, Liu CJ (2012). Asian-Pacific consensus statement on the management of chronic hepatitis B: a 2012 update.. Hepatol int..

[2] You S, Rong Y, Zhu B, Zhang A, Zang H, Liu H (2013). Changing etiology of liver failure in 3,916 patients from northern China: a 10-year survey.. Hepatol Int..

[3] Wlodzimirow KA, Eslami S, Abu-Hanna A, Nieuwoudt M, Chamuleau RA (2013). A systematic review on prognostic indicators of acute on chronic liver failure and their predictive value for mortality.. Liver Int..

[4] Zheng YB, Huang ZL, Wu ZB, Zhang M, Gu YR, Su YJ (2013). Dynamic changes of clinical features that predict the prognosis of acute-on-chronic hepatitis B liver failure: a retrospective cohort study.. Int J Med Sci..

[5] Jalan R, Gines P, Olson JC, Mookerjee RP, Moreau R, Garcia-Tsao G (2012). Acute-on chronic liver failure.. J Hepatol..

[6] Peng L, Xie DY, Lin BL, Liu J, Zhu HP, Xie C (2011). Autologous bone marrow mesenchymal stem cell transplantation in liver failure patients caused by hepatitis B: short-term and long-term outcomes.. Hepatology..

[7] Cui YL, Yan F, Wang YB, Song XQ, Liu L, Lei XZ (2010). Nucleoside analogue can improve the long-term prognosis of patients with hepatitis B virus infection-associated acute on chronic liver failure.. Dig Dis Sci..

[8] Zheng Z, Li X, Li Z, Ma X (2013). Artificial and bioartificial liver support systems for acute and acute-on-chronic hepatic failure: A meta-analysis and meta-regression.. Exp Ther Med..

[9] Zhang GL, Xie DY, Lin BL, Xie C, Ye YN, Peng L (2013). Imbalance of interleukin-17-producing CD4 T cells/regulatory T cells axis occurs in remission stage of patients with hepatitis B virus-related acute-on-chronic liver failure.. J Gastroenterol Hepatol..

[10] Fujiwara K, Yasui S, Yonemitsu Y, Mikata R, Arai M, Kanda T (2014). Efficacy of high-dose corticosteroid in the early stage of viral acute liver failure.. Hepatol Res..

[11] Fujiwara K, Yasui S, Yonemitsu Y, Fukai K, Arai M, Imazeki F (2008). Efficacy of combination therapy of antiviral and immunosuppressive drugs for the treatment of severe acute exacerbation of chronic hepatitis B.. J Gastroenterol..

[12] Sarin SK, Kumar A, Almeida JA, Chawla YK, Fan ST, Garg H (2009). Acute-on-chronic liver failure: consensus recommendations of the Asian Pacific Association for the study of the liver (APASL).. Hepatol Int..

[13] Sheen IS, Liaw YF, Tai DI, Chu CM (1985). Hepatic decompensation associated with hepatitis B e antigen clearance in chronic type B hepatitis.. Gastroenterology..

[14] Sun QF, Ding JG, Xu DZ, Chen YP, Hong L, Ye ZY (2009). Prediction of the prognosis of patients with acute-on-chronic hepatitis B liver failure using the model for end-stage liver disease scoring system and a novel logistic regression model.. J Viral Hepat..

[15] Matsumoto K, Miyake Y, Miyatake H, Takahara M, Imada T, Yagi S (2009). A combination treatment of entecavir and early-phase corticosteroid in severe exacerbation of chronic hepatitis B.. World J Gastroenterol..

[16] Zou Z, Li B, Xu D, Zhang Z, Zhao JM, Zhou G (2009). Imbalanced intrahepatic cytokine expression of interferon-gamma, tumor necrosis factor-alpha, and interleukin-10 in patients with acute-on-chronic liver failure associated with hepatitis B virus infection.. J Clin Gastroenterol..

[17] Hu X, Ma S, Huang X, Jiang X, Zhu X, Gao H (2011). Interleukin-21 is upregulated in hepatitis B-related acute-on-chronic liver failure and associated with severity of liver disease.. J Viral Hepat..

[18] Shen C, Yan WZ, Zhao CY, Che HH, Liu XY, Liu ZZ (2013.). Increased CD4CD25 regulatory T cells correlate with poor short-term outcomes in hepatitis B virus-related acute-on-chronic liver failure patients.. J Microbiol Immunol Infect..

[19] Das A, Ellis G, Pallant C, Lopes AR, Khanna P, Peppa D (2012). IL-10-producing regulatory B cells in the pathogenesis of chronic hepatitis B virus infection.. J Immunol..

[20] Turner DM, Williams DM, Sankaran D, Lazarus M, Sinnott PJ, Hutchinson IV (1997). An investigation of polymorphism in the interleukin-10 gene promoter.. Eur J Immunogenet..

[21] Sofian M, Kalantar E, Aghakhani A, Hosseini S, Banifazl M, Eslamifar A (2013). No correlation between interleukin-10 gene promoter polymorphisms and hepatitis B virus infection outcome.. Hepat Mon..

[22] Nelson DR, Lauwers GY, Lau JY, Davis GL (2000). Interleukin 10 treatment reduces fibrosis in patients with chronic hepatitis C: a pilot trial of interferon nonresponders.. Gastroenterology..

[23] Nelson DR, Tu Z, Soldevila-Pico C, Abdelmalek M, Zhu H, Xu YL (2003). Long-term interleukin 10 therapy in chronic hepatitis C patients has a proviral and anti-inflammatory effect.. Hepatology..

[24] Sagnelli E, Manzillo G, Maio G, Pasquale G, Felaco FM, Filippini P (1980). Serum levels of hepatitis B surface and core antigens during immunosuppressive treatment of HBsAg-positive chronic active hepatitis.. Lancet..

[25] Karkhanis J, Verna EC, Chang MS, Stravitz RT, Schilsky M, Lee WM (2014). Steroid use in acute liver failure.. Hepatology..

